# Bilateral Primary Total Knee Arthroplasty and Reconstruction of the Medial Tibial Plateau by an Asymmetric Cone in a Patient with Charcot Arthropathy

**DOI:** 10.1155/2021/9965640

**Published:** 2021-06-08

**Authors:** Spiros Tsamassiotis, Henning Windhagen, Max Ettinger

**Affiliations:** Hannover Medical School, Department of Orthopaedic Surgery, Anna-Von-Borries Str. 1-7, 30625 Hannover, Germany

## Abstract

Charcot arthropathy of the knee is an extremely rare orthopedic disease that is very challenging for the treating physician and is associated with many complications, especially if it is occurring on both knees. Meanwhile, in the advanced stage, despite many potential complications, TKA is recognized as the gold standard. However, destruction of the medial tibial plateau is typical for the disease, which makes a stable anchorage of the prosthesis much more difficult. Therefore, we present a case in which sufficient primary stability could be achieved with an asymmetrical second-generation tibial cone with an anatomical design and implantation instruments adapted to the bony anatomy in the presence of severe tibial destruction on both sides. In the two-year follow-up, the patient showed good mobility and stability on both sides. In advanced Charcot arthropathy of the knee, the use of asymmetric tibial cones appears to be an appropriate solution for secure fixation and stability of the implant.

## 1. Introduction

Charcot arthropathy is a rare and challenging clinical picture for orthopedic surgeons. Formerly most commonly associated with tabes dorsalis in the context of syphilis, Charcot arthropathy is now, with respect to the invention of penicillin, most often associated with the widespread disease diabetes mellitus (DM) [1].

This disease is characterized by rapid joint destruction. An inadequately functioning proprioception and nociception lead to repetitive microtraumas [2]. In addition, a dysfunction of the sympathetic nervous system causes increased blood flow in the bones as well as disturbed bone metabolism. This leads to osteolysis and increased bone resorption [1, 3]. The pathomechanism of the disease is highly complex and involves a variety of neuronal transmitters and neurotrophic factors elaborated in bone formation, resorption, and remodelling [4].

Charcot arthropathy, which is rare overall, usually manifests itself in the area of the foot and ankle joints [5]; Charcot arthropathy of the knee joint is a rarity [6]. For a long time, arthrodesis was regarded as the therapy of choice and total knee arthroplasty (TKA) as a contraindication due to the many serious complications that can occur in Charcot arthroplasty of the knee [7–9]. Due to the low patient acceptance of arthrodesis and poor functional results, knee endoprostheses have been implanted with increasing frequency in selected Charcot patients in recent years [10]. The medium-term results were satisfactory [8].

As with any knee endoprosthetic procedure, the restoration of the joint line and balanced flexion and extension gaps guarantee a good functional result. However, the implantation of a knee endoprosthesis in a Charcot situation is by no means comparable to a primary TKA. On the contrary, such an intervention shows many parallels to revision knee arthroplasty. In addition to the often very poor bone quality, advanced destruction is often found in Charcot knees in an overall unstable situation. Typical for a Charcot knee is a severely destroyed or no longer present medial tibial plateau. Thus, a bone defect can result in severe varus and lead to ligamentary instability. The AORI classification can be used to classify the bone defects of Charcot knees [11].

In addition to the classical methods such as cementation, augmentation, or the use of various autologous or allogenic bone grafting techniques to bridge such defects, sleeves and cones are becoming increasingly popular [12]. An alleged advantage of cones over sleeves is their metaphyseal fixation independent of the component or stem, since they are not coupled to the component or stem-like sleeves. This means that the joint line can be restored and the flexion and extension gap can be balanced out before the defect is bridged. In the past, tantalum cones were used in a few cases for Charcot knees [13]. These first-generation cones require preparation of the bone bed with high-speed drills according to the trial and error principle until the cone and bone bed fit together exactly [14]. However, such a preparation can lead to fractures, especially in sclerotic bone. A fracture set intraoperatively by the surgeon in a Charcot knee that already suffers from severe bone defects with possible additional ligamentary instability can have devastating consequences. In contrast, in second-generation cones, a streamlined reamer-based instrumentation replaces high-speed drilling. In addition, the asymmetric, anatomical design of the cone fits well with the extended destruction of the medial tibial plateau, typical in Charcot knees. Therefore, it is the purpose of this case report to present a Charcot case in which a bilateral asymmetric bone defect of the tibial plateau was bridged by a second-generation asymmetric tibial cone. TKA for Charcot arthropathy is rare, and bilateral occurrence is even more. As far as we know, after an intensive study of the relevant literature, we could not find any case report in which asymmetric tibial cones were used for bilateral Charcot arthropathy.

## 2. Case Presentation

In November 2018, a 58-year-old female patient with bilateral knee joint complaints was referred to the outpatient clinic. At this time, the patient could only mobilize herself in a wheelchair because of the pain and was wearing knee braces to stabilize the knees.

The patient had previously undergone an open reconstruction of the left foot with tibiocalcaneal arthrodesis in our clinic using an external fixator because of Charcot arthropathy, Sanders IV, and Eichenholtz II with open ankle dislocation. On the right foot, the patient suffers from a pes planovalgus malalignment with charcoid bone condition. The patient was mobilized in orthopedic boots until the onset of the knee joint complaints and coped very well with them. The patient was 170 cm tall and weighed 115 kg (BMI 39.8). The patient suffered from insulin-dependent DM type II.

The physical examination showed a severe varus malalignment of the knee with increased medial laxity. The antero-posterior stability could not be reliably checked due to severe pain. The active range of motion (ROM) was extension/flexion 5/0/80° for the right knee and 0/0/85° for the left knee.

**Figure 1:**
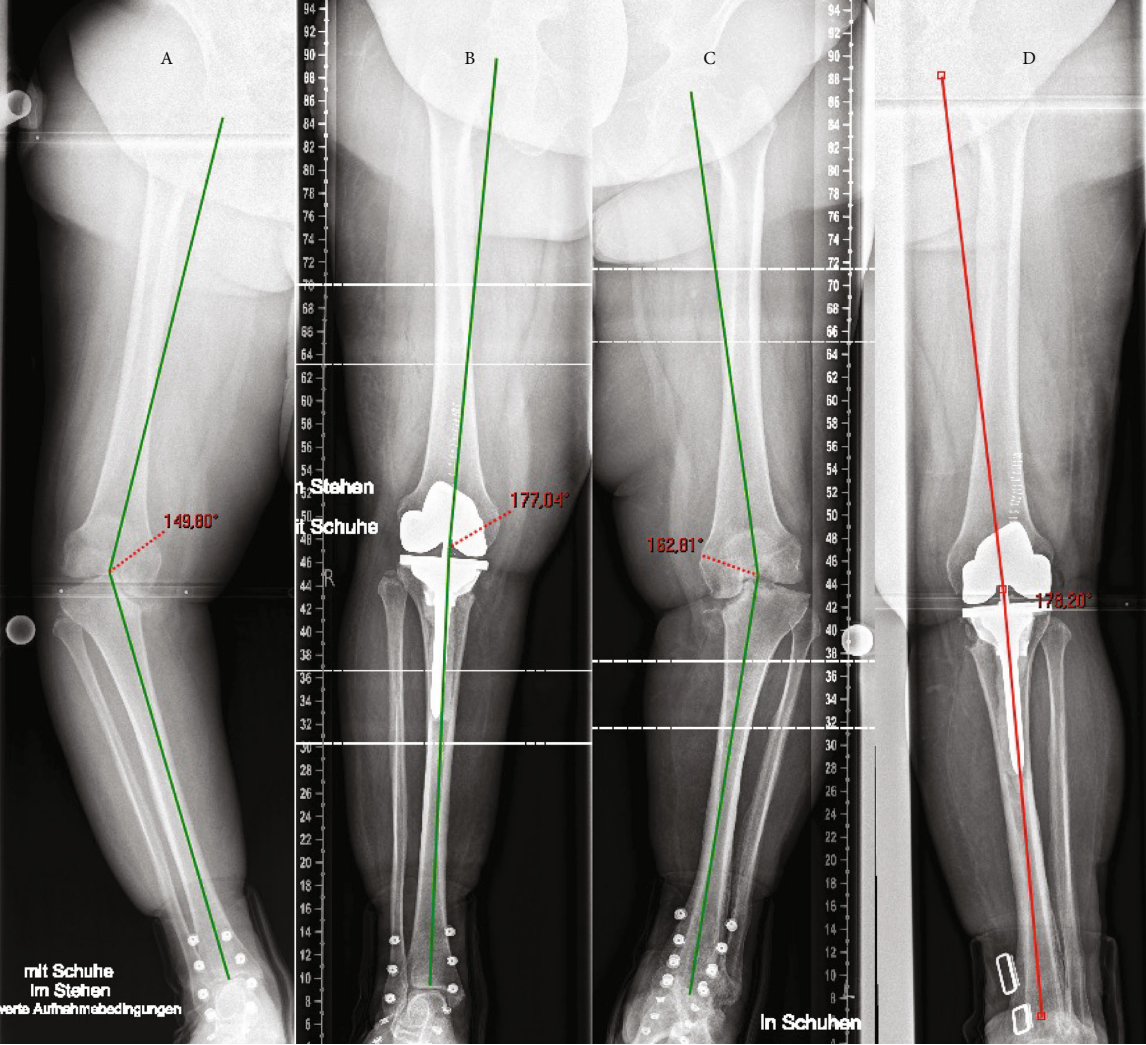
Standing full leg axis radiographs in orthopedic boots of both sides show a severe varus deformation of about 30° (a) and 17° (c) and the postoperative outcome with an almost neutral leg axis (b, d).

Preoperative standing full leg X-rays demonstrate a severe varus malalignment of 30° right and 17° left (Figures 1(a) and 1(c)). Both medial tibial plateaus were almost completely impacted with only minimal bone remaining.

**Figure 2:**
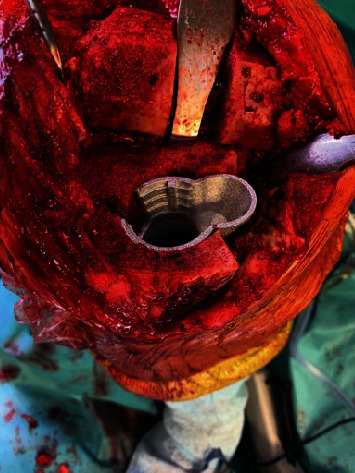
Intraoperative situation after insertion of the cone. Note how the asymmetric design bridges the extensive medial bone defect.

**Figure 3:**
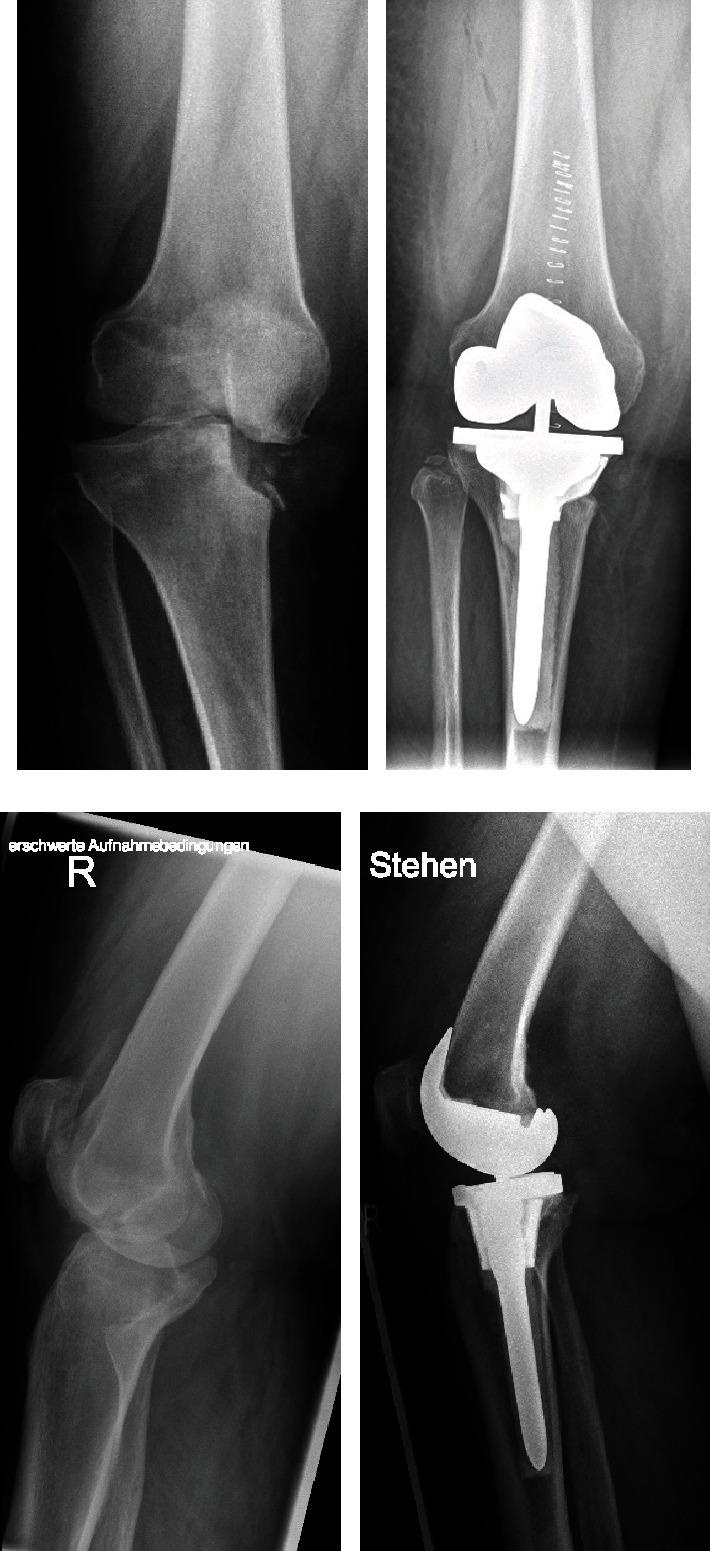
Gonarthritis IV° and completely impacted medial tibial plateau with small diffuse bony remains (a, c). Postoperative standing antero-posterior and lateral X-ray of the right knee. Pay attention to the asymmetric tibial cone which reconstructs the medial tibial plateau (b, d).

The right knee was operated in November 2018 and the left knee in January 2019. A continuous femoral nerve block (cFNB) was executed preoperatively. The medial parapatellar access was used for prosthesis implantation. A femur first mechanical alignment approach was conducted. IV° osteoarthritis was found in the patellofemoral and lateral compartment. The anterior cruciate ligaments were frayed and insufficient. The medial collateral ligaments presented to be sufficiently stable under valgus stress, but the lateral collateral ligaments (LCL) were stretched out. The Stryker Triathlon® TS prosthesis system (Stryker, Kalamazoo, MI, USA) was used. An asymmetrical cone was used to reconstruct the medial tibial plateau (Figure 2). The bone-cone interface is a press fit anchorage. The cone-prosthesis interface is connected by cement. The prosthesis itself is cemented, and the stem is linked to the tibial component. To ensure sufficient multidirectional stability, a total stabilized (TS) implant was used to compensate for the stretched out LCL. Using a balancer, the extension and flexion gaps were checked for symmetry. For immediately sufficient postoperative pain control, the patient received local infiltration analgesia additionally. No drains were used. The direct postoperative ROM under general anaesthesia was 0/0/120° for extension/flexion. The cFNB remained for three days. Sufficient pain control for adequate mobilization could be achieved. Full loading was allowed immediately. The patient remained in the hospital for 11 days after the operation and was then discharged to her home after the right knee. The patient was discharged after 9 days on the left side. On discharge, the patient was mobile with crutches with an active ROM of extension/flexion 0/0/90°. The postoperative X-rays with an almost neutral leg axis (remaining varus of 3° right and 2° left) are shown in Figures 1(b) and 1(d).  Figure 3 shows a close-up of the pre- and postoperative status of the right knee in two planes.

After a two-year follow-up, the patient suffered no pain and was mobile with one crutch due to her comorbidities. Both knees show an active ROM of 0/0/115 in combination with full varus-valgus stability.

## 3. Discussion

This two-year follow-up demonstrates a good clinical result for severe neuropathic arthropathy. A neuropathic arthropathy, e.g., in the context of DM, represents a great challenge for the surgeon due to the disturbed bone metabolism with bone loss, poor bone quality, and ligament laxity.

TKA in such cases is a procedure described and acknowledged in the current literature [13]. Nevertheless, TKA in Charcot knees is associated with a high complication rate [8, 15]. In particular, the frequently seen massive defect of the medial tibial plateau must be bridged, and the knee, which is often unstable, must be adequately stabilized, also regarding the postoperative phase with impaired sensorimotor function [5, 13]. There is disagreement in the literature about which degree of constraint should be used in Charcot knees. Some authors argue against the use of higher degrees of constraint because of the increased stress in the bone-cement and cement-implant interface, possible fractures, and increased bone resection [13]. In particular with rotating hinge models, the loosening rate and the risk of fracture are often used as arguments against such prostheses, while the increased degree of stability is used as an argument in favour of these prostheses [15]. Other authors, however, were able to present similar results with rotating hinge prostheses as with primary TKA [16]. Nevertheless, many patients who have been treated with a rotating hinge knee still need a walking aid after 10 years [17].

In our opinion, the choice of the degree of constraint should be based not only on the bony defect but also on a mandatory intraoperative assessment of the ligament status. If the collateral ligaments are partially affected, a TS implant can also be used in Charcot knees as in the case presented here. In the case described, there was a multiligamentous instability with a stretched out LCL. Therefore, a TS implant was used which offers ±3° varus/valgus stability. Revision surgery with TS implants can achieve similar postoperative results as primary TKA, so that one can assume a high postoperative functionality with a TS implant [18]. In the case presented, the patient was able to extend her knees 0° and bend them 90° at discharge. A tibial stem must be implanted with a TS implant to ensure adequate force application that does not jeopardise the prosthesis durability.

The second challenge in Charcot knee is bridging the extensive bone defects which do not easily allow metaphyseal fixation of the implant in zone two. In our case study, as is the case in most Charcot knees, in addition to the massive arthritic changes of the lateral and patellofemoral compartments, the medial tibial plateaus were almost completely destroyed. To bridge such large structural defects, structural allografts, modular metal augments, sleeves or, as in the case described here, cones can be used [19]. Despite good short- and medium-term clinical results with allografts and augments, complications can occur with both procedures. Structural allografts require a long stem, which can lead to stem pain and stress shielding. Moreover, the allograft may resorb or collapse. Furthermore, there is a risk of infection and there may be insufficient remodelling and revascularization. Modular metal augments are generally used in an older and more inactive patient population. However, Charcot arthropathy also affects young patients. In addition, there is a risk of friction, corrosion, stress shielding with bone loss, and the formation of radiolucent lines, and the application is problematic in case of severe cancellous bone damage. Also, many patients complain of dissatisfaction and pain after metal augmentation [12, 19–27]. Sleeves can be inserted both cemented and uncemented. Just like cones, they are fixed in zone two and provide reduced stress shielding with increased rotational stability. With both options, the surgeon has better control over the rotational alignment and can restore the joint line. A shorter stem can be used with both options. With sleeves, a stem can be omitted, but the postoperative outcomes are sometimes not good. The disadvantage of both is the relatively high costs. Both implants achieved satisfactory short-term results. Sleeves are firmly connected to the implant via a morse taper junction and are therefore implant-specific, while cones are connected via a cement interface. Both connections are a potential source of failure. Both options are relatively difficult to remove in case of revision. Sleeves often require an osteotomy. In general, however, cones have a higher modularity than sleeves. Nevertheless, high fracture rates have been reported for first-generation tantal cones, and there is a risk of avulsion of the patella tendon. In addition, bone preparation is sometimes challenging [12, 14, 28–30]. An intraoperative fracture in the case of a Charcot knee can result in catastrophic consequences.

Therefore, in the case described here, a second-generation cone was used, as the reconstruction of the medial tibial plateau was crucial. The used cone has an anatomical design based on a CT database. The instrumentation required for bone preparation is also adapted to the bony anatomy. A streamlined reamer-based instrumentation replaces the manual high-speed drilling of the first generation and provides immediate press fit [31, 32]. In addition, the asymmetric variant of the tibial cone used in this case enables a sufficient and primary stable reconstruction of the bone defect of the medial tibial plateau, which is typical for Charcot knees. The asymmetric tibial cone also offers the surgeon up to 10° of rotational freedom and is characterized by good accuracy of fit. Under physiological load, these cones, made of porous titanium, are at least equivalent and in some cases superior to tantal cones in terms of mechanical stability [31, 32].

In summary, the adequate endoprosthetic treatment of a Charcot knee is a great challenge for the surgeon. Both instabilities and extensive bony defects have to be addressed. In the case presented here, the Charcot-typical bone defect of the medial tibial plateau could be excellently addressed bilaterally with the use of an asymmetrical second-generation tibial cone. There was no need to resort to a hinged prosthesis model or to use structural allografts or augments bilaterally. However, long-term results of the second-generation metaphyseal cones must be awaited.
